# The Use of Cocaine by Dentists

**Published:** 1907-01-19

**Authors:** Frederick B. Penfold


### THE USE OF COCAINE BY DENTISTS.

By Frederick B. Penfold, M.R.C.S., L.R.C.P.Lond., L.D.S., R.C.S.Eng.

A Correspondent writes: "Numerous dentists are quite willing to inject into the gum a mixture containing cocaine, even when they know the patient to be suffering from heart disease, and yet medical men in such cases emphatically refuse to sanction such administration, even for the extraction of a single tooth. If there have been fatalities directly resulting from these injections, the large number of sufferers from heart disease who innocently request the dentist to use the drug should surely be cautioned. At the same time it would be interesting to know if there exists any local anæsthetic safe and efficient."

The inquiry whether "numerous dentists are quite willing to inject into the gum a mixture containing cocaine" can be met with an affirmative answer, and it may be said that this humane form of treatment has been carried on successfully for many years, rendering bearable what is acknowledged to be an excruciatingly painful operation. Cocaine, or its derivative eucaine, in combination with other drugs, is in daily use by dentists for many painful operations, apart from extraction, and untoward results, much less fatalities, are very few and far between. One would have to spend a deal of time to find records of fatalities resulting directly from these injections performed by a qualified dentist on a normal subject.

It is as well to compare the dosage of these "mixtures containing cocaine" with that of the preparations given in the British Pharmacopœia, and it will be seen that the druggists have minimised to the lowest point consistent with efficiency the percentage of cocaine contained in these mixtures. While the British Pharmacopœia allows a dose of 2 to 5 min. of a 10 per cent. solution of cocaine in the official hypodermic preparation—the proprietary preparations are usually 1 per cent., and often less. When eucaine is used the percentage is still lower. So that these mixtures containing cocaine are not so deadly as one would suppose at first sight, and after many years of experience the druggist has obtained an almost non-toxic preparation which contains less than 1 per cent. cocaine.

To minimise general absorption, and to produce a more complete local anæsthesia, many drugs have been resorted to in combination with cocaine or its derivatives. Of these the most important is suprarenal extract, which is used with the objects above named, and for its hæmostatic properties. The cases where untoward results have attended a cocaine injection are not numerous, and as a rule the symptoms do not last more than a few minutes. It is not uncommon to have the same condition occur after an extraction conducted without the help of a local anæsthetic, in which case the shock is far greater and the operation far more trying to the patient.

There does not seem any indication which will lead one to foretell the cases in which cocaine will act adversely, but such a result occurs more often in the young; and strong, healthy individuals are as often affected as the weakly. Now and again an apparently healthy individual will show some untoward symptoms, such as slight faintness and pallor, with perspiration and giddiness, all of which pass off in a few minutes. Occasionally sickness may follow, and this usually terminates a series of symptoms, which are quite as likely to occur after an extraction without cocaine.

Although occasionally, serious symptoms do arise from small doses of cocaine, they can justly be put down to personal "idiosyncrasy," in the same way that chloroform affects, in some unusual manner, certain individuals. One must not imagine because there is no means of foretelling such an idiosyncrasy that patients should be left to providence to protect them. It is the duty of every professional man to treat each patient as though the subject of an idiosyncrasy, and to proceed with the injection cautiously, little by little, until the whole area is sufficiently anæsthetised. It would be courting the disaster of publicity in a coroner's court for a dentist to administer cocaine if he is aware that the patient's medical adviser has emphatically refused to sanction such administration.

A dentist after his five years' training, which includes a course of dental therapeutics, is quite aware of the action of cocaine as he is of many other substances, poisonous and otherwise, which he has to use, and he is quite prepared with the usual antidotes and restoratives in the case of accident. In using cocaine he must of course take all precautions, and if, for any reason, the advisability of its use in an individual case is a matter of doubt, he should, prior to the administration of the drug, take the opinion of the patient's medical adviser. But for a dental practitioner to discuss with his patient the safety, or otherwise, of cocaine as a local anæsthetic, is neither necessary nor advisable.

